# The impact of early factors on persistent negative symptoms in youth at clinical high risk for psychosis

**DOI:** 10.3389/fpsyt.2023.1125168

**Published:** 2023-05-24

**Authors:** Daniel J. Devoe, Lu Lui, Tyrone D. Cannon, Kristin Suzanne Cadenhead, Barbara A. Cornblatt, Matcheri Keshavan, Tom H. McGlashan, Diana. O. Perkins, Larry J. Seidman, William S. Stone, Ming T. Tsuang, Scott W. Woods, Elaine F. Walker, Daniel H. Mathalon, Carrie E. Bearden, Jean Addington

**Affiliations:** ^1^Department of Psychiatry, Hotchkiss Brain Institute, University of Calgary, Calgary, AB, Canada; ^2^Department of Psychology, Mount Royal University, Calgary, AB, Canada; ^3^Department of Psychology, Yale University, New Haven, CT, United States; ^4^Department of Psychiatry, University of California, San Diego, San Diego, CA, United States; ^5^Department of Psychiatry, Zucker Hillside Hospital, Queens, NY, United States; ^6^Department of Psychiatry, Harvard Medical School at Beth Israel Deaconess Medical Center and Massachusetts Mental Health Center, Boston, MA, United States; ^7^Department of Psychiatry, Yale University, New Haven, CT, United States; ^8^Department of Psychiatry, University of North Carolina, Chapel Hill, NC, United States; ^9^Institute of Genomic Medicine, University of California, San Diego, San Diego, CA, United States; ^10^Department of Psychology, Emory University, Atlanta, GA, United States; ^11^Department of Psychiatry, University of California, San Francisco, San Francisco, CA, United States; ^12^Psychiatry Service, San Francisco, CA, United States; ^13^Department of Psychiatry and Biobehavioral Sciences, University of California, Los Angeles, Los Angeles, CA, United States; ^14^Department of Psychology, University of California, Los Angeles, Los Angeles, CA, United States

**Keywords:** persistent negative symptoms, clinical high risk, premorbid functioning, psychosis, trauma, life events, cannabis, early factors

## Abstract

**Introduction:**

Persistent negative symptoms (PNS) are described as continuing moderate negative symptoms. More severe negative symptoms have been associated with poor premorbid functioning in both chronic schizophrenia and first episode psychosis patients. Furthermore, youth at clinical high risk (CHR) for developing psychosis may also present with negative symptoms and poor premorbid functioning. The aim of this current study was to: (1) define the relationship between PNS and premorbid functioning, life events, trauma and bullying, previous cannabis use, and resource utilization, and (2) to examine what explanatory variables best predicted PNS.

**Method:**

CHR participants (*N* = 709) were recruited from the North American Prodrome Longitudinal Study (NAPLS 2). Participants were divided into two groups: those with PNS (*n* = 67) versus those without PNS (*n* = 673). A K-means cluster analysis was conducted to distinguish patterns of premorbid functioning across the different developmental stages. The relationships between premorbid adjustment and other variables were examined using independent samples t-tests or chi square for categorical variables.

**Results:**

There was significantly more males in the PNS group. Participants with PNS had significantly lower levels of premorbid adjustment in childhood, early adolescence, and late adolescence, compared to CHR participants without PNS. There were no differences between the groups in terms of trauma, bullying, and resource utilization. The non-PNS group had more cannabis use and more desirable and non-desirable life events.

**Conclusion:**

In terms of better understanding relationships between early factors and PNS, a prominent factor associated with PNS was premorbid functioning, in particular poor premorbid functioning in later adolescence.

## 1. Introduction

Negative symptoms are a substantial cause of burden for patients with psychosis and their caregivers, impacting both functioning and quality of life (1, 2), and result in increased healthcare resource utilization and costs (3). However, negative symptoms have also been observed in those at clinical high risk (CHR) for psychosis (4, 5). Severity of negative symptoms in those at CHR have associations with poor outcomes such as functional deficits (6), social difficulties (7), and transition to psychosis (4). Moreover, negative symptoms may be multidimensional and be associated with cognitive impartments (8, 9). One area that has remained understudied in CHR youth involves persistent negative symptoms (PNS), which are defined here as clinically stable negative symptoms of moderate severity evident for an extended period of time. Exploration of early factors that may contribute to PNS in CHR youth is warranted, as it may help us understand the early determinants of negative symptoms. Early factors that could potentially be associated with PNS include premorbid functioning, early trauma, life events, and cannabis use.

CHR youth frequently exhibit poor premorbid functioning compared to healthy controls (10, 11), and demonstrate premorbid functioning akin to patients with psychosis (12). Moreover, studies have shown that poorer premorbid functioning in CHR youth is significantly correlated with worse negative symptom severity in late adolescence (13, 14). In one CHR study, a cluster analysis demonstrated that a deteriorating pattern of functioning was associated with worse negative symptoms and poorer social functioning relative toto stable-intermediate and stable-good patterns of functioning (13). In one study, premorbid social adjustment was significantly worse in the PNS group compared to those without PNS for both early and late adolescences, and academic adjustment in late adolescence (5).

In a recent meta-analysis, childhood trauma was significantly more prevalent in CHR youth compared to healthy controls (15). A longitudinal study demonstrated that individuals at CHR for psychosis reported significantly more trauma and bullying than healthy controls, and associations between bullying and negative symptoms (16). However, two studies to date have demonstrated no relationship between childhood trauma and negative symptoms in CHR youth (16, 17), leading to the conclusion in a recent review that the association between early trauma and negative symptoms in CHR youth remains inconclusive (18). Since bullying has been linked with negative symptoms and the association with trauma remains inconclusive, it may be important to investigate the relationship with trauma and bullying in CHR youth who present with more pronounced negative symptoms such as PNS.

Another meta-analysis demonstrated that life-event rates were significantly lower in CHR youth compared to healthy controls (15), which has led to speculation that a potential explanation for lower life-event rates in CHR, involves negative symptoms such as increased avolition and social withdrawal, which ultimately leads to fewer life events (18, 19).

To date, only a few studies have addressed the relationship between negative symptoms and cannabis use in CHR participants (20–22). Total negative symptoms at baseline has not been linked with previous cannabis use (20–22); however, one study found that current weekly cannabis users had higher total negative symptoms (23). A recent review reported no significant difference between cannabis users and non-users on total negative symptoms (24).

Thus, exploration of early factors in a large CHR longitudinal cohort may provide greater insight into the determinants of PNS. Determining whether PNS in CHR youth is related to premorbid functioning, desirable and undesirable life events, trauma and bullying, and previous cannabis use may provide greater insights into the detectability and trajectory of those who go on to develop PNS.

### 1.1. Aims

The present study examined PNS in CHR youth in a large longitudinal cohort [North American Prodrome Longitudinal Study (NAPLS 2)] (25). The aim of this current study was to: (1) define the relationship between PNS and premorbid functioning, desirable and undesirable life events, trauma and bullying, previous cannabis use, and resource utilization; and (2) to examine what explanatory variables best predicted PNS. We hypothesized that CHR youth with PNS would show significant deficits in premorbid functioning, have experienced more trauma and bullying, have a greater history of cannabis usage, present with more undesirable life events, and have more healthcare resource utilization compared to CHR participants without PNS. Furthermore, it was hypothesized that poor premorbid functioning would be the strongest predictor of future PNS.

## 2. Methods

### 2.1. Participants

CHR participants (*N* = 764; 436 males, 328 females) between the ages of 12 and 35 years old were recruited as part of the 8-site North American Prodrome Longitudinal Study (NAPLS-2). Participants were referred to NAPLS-2 by health care providers, educators, social service agencies, or were self-referred in response to extensive community education efforts. Potential participants underwent a screen and those who screened positive were subsequently invited to an in-person eligibility evaluation and consent (25). At baseline, 743 participants met CHR criteria using the Criteria of Psychosis-risk Syndromes (COPS) based on the Structured Interview for Psychosis-risk Syndromes (SIPS) (26). Twenty-one participants were considered high risk because they were under the age of 19 and presented with schizotypy. Exclusion criteria were an IQ <70, any axis I current or lifetime psychotic disorder, past or current history of a central nervous system disorder, and substance dependence in the 6-months prior to enrollment. A more detailed description of the inclusion and exclusion criteria and study measures are described elsewhere (25, 27).

For this study, we included CHR participants who had negative symptom data at both baseline and follow-ups in order to determine the presence of PNS. Twenty-four participants did not have sufficient negative symptom data at baseline, leaving a sample of 740 CHR participants. We included all CHR subjects with negative symptoms who met criteria for PNS (*n* = 67), as defined below, or who did not meet criteria; non-PNS (*n* = 673).

### 2.2. Procedures

The study was approved by institutional review boards at all NAPLS-2 sites. All participants provided written informed consent, including parental consent. Trained raters conducted clinical assessments at baseline, 6, 12, 18, and 24 months. Intraclass correlations for the Scale of Psychosis-risk Symptoms (SOPS) total scores were in the excellent range (27).

### 2.3. Assessments

Negative symptoms were rated on the SOPS (26). For the current study, SOPS negative symptoms were restricted to social anhedonia (N1), avolition (N2), and expression of emotion (N3) to align with the NIMH-MATRICS negative symptom consensus on current domains for negative symptoms (2).

Participants were rated on premorbid functioning with the Premorbid Adjustment Scale (PAS) (28). The PAS measures premorbid functioning in four areas of development; sociability and withdrawal, peer relationships, scholastic performance, adaption to school, and socio-sexual aspects of life. The areas of development are measured for each of the four developmental stages of childhood (up to age 11), early adolescence (12–15 years), late adolescence (16–18 years), and adulthood (19 and up) (29). Only three developmental stages, including childhood, early adolescence, and late adolescence from the PAS were utilized in all statistical analyzes. This was due to the young age of the current sample, which is reflected by the small proportion of completed adulthood subscales.

Previous experience of trauma and abuse was assessed using the Childhood Trauma and Abuse Scale (30). In a semi-structured interview, participants were asked about emotional, physical, psychological, and sexual abuse that occurred before the age of 16. A total trauma score out of four was generated to include the sum of emotional, physical, psychological, and sexual abuse. In addition, participants were asked if they had experienced bullying, either physical, or psychological or both. Participants were considered to have experienced bullying if they reported physical, psychological or both.

A modified version of the Psychiatric Epidemiology Research Interview Life Events Scale was administered (31). The life events scale was modified to exclude items irrelevant to adolescents in this study (e.g., getting a divorce), for a total of 59 included items pertaining to significant events or life changes that could have occurred at any point in their life. Life events items are designated as independent or dependent and are also classified as desirable or undesirable experiences. The subjective stress for each life event endorsed was rated on a 7-point Likert scale ranging from “occurred but was not very stressful” to “caused me to panic.”

A cannabis scale based on commonly used measures and interview questions in the literature (32) was used to record the history of cannabis usage. Participants were asked about total usage in their lifetime, past or current usage, age of first usage, and frequency of usage.

Participants were asked about previous resource utilization for emergency visits due to physical problems, emergency visits for psychiatric problems, inpatient visits for physical problems, inpatient visits for psychiatric problems, and day hospitalizations.

### 2.4. Definition of persistent negative symptoms

PNS were defined as having one of the following three negative symptoms: social anhedonia (N1), avolition (N2), and expression of emotion (N3) scored ≥4 (i.e., moderately severe to extreme) for a duration of 1 year.

### 2.5. Analyzes

Participants were divided into two groups, the PNS group versus the non-PNS group. Chi square tests were used to compare the groups on gender, cannabis usage, and resource utilization, and Mann–Whitney U tests were used for comparison of total trauma and total bullying. Independent t-tests were used to compare the differences between the groups on premorbid functioning variables and age.

Generalized linear mixed models for repeated measures were utilized to examine changes over time (i.e., baseline, 12, and 24 months) on the life events scale between and within groups to accommodate for missing data and account for intra-participant correlations.

Cluster analysis was used to identify distinct patterns of premorbid functioning. K-means cluster analysis was used to assign cases to a fixed number of groups (clusters). This procedure attempts to identify relatively homogeneous groups of cases based on selected characteristics using an algorithm that can handle a large number of cases. The algorithm requires pre-specification of the number of clusters. To classify cases, we updated cluster centers iteratively. We used the PAS developmental subscale scores for all 709 subjects (who had completed the PAS) in the analysis. The decision to use a k-means cluster method was based on past research done in the area and the potential of this analysis to handle missing cases. This was of particular importance given the developmental nature of the PAS and the variation in the time frame for determining an individuals’ premorbid functioning.

For the prediction of PNS, a full logistic regression model was built with all explanatory variables. Variable inclusion and selection were made by dropping the variables with less significance one by one, creating a final model with one predictor of the binary dependent variable.

All statistical tests were 2-sided and a *p* value of <0.05 was considered statistically significant. All tests were adjusted for multiple comparisons using Tukey–Kramer. Analyzes were performed using both IBM SPSS Statistics for Mac version 25 and SAS version 9.2 (33).

## 3. Results

Of 740 CHR participants, 67 (9.05%) had PNS and 673 (90.95%) did not. There were significantly more males in the PNS group [*x*^2^ (1) = 6.19; *p* = 0.01]; there was a total of 48 males in the PNS group and 376 in the non-PNS group. However, the groups did not differ in age, the PNS mean age was 18.5 and the non-PNS mean age 18.7.

### 3.1. Differences in trauma and bullying

No significant differences were found between the groups for rates of total trauma or bullying.

### 3.2. Differences in life events

Generalized linear mixed models demonstrated the non-PNS group had significantly more total desirable events than PNS group over time, the non-PNS group had significantly more total dependent events than PNS group at baseline and 12 months, and the non-PNS group had significantly more total undesirable events than PNS group at baseline (See Table 1).

**Table 1:** (a) Differences in life events between groups. (b) Differences in life events within groups.

(a)						
	PNS (*n* = 67)			NON-PNS (*n* = 672)		
Life events	Mean (SE)					
	Baseline	12 months	24 months	Baseline	12 months	24 months
Total dependent	12.3 (0.79)	5.1 (0.59)	4.6 (0.7)	15.4 (0.27)a**	7.8 (0.26)b***	7.6 (0.31)
Total independent	2.9 (0.22)	0.4 (0.15)	0.5 (0.18)	3.3 (0.07)	0.8 (0.07)	0.9 (0.08)
Total desirable	5.0 (0.28)	2.8 (0.25)	2.8 (0.28)	6.4 (0.09)a***	4.3 (0.10)b***	4.2 (0.1)c***
Total undesirable	8.1 (0.62)	2.6 (0.44)	2.2 (0.52)	9.9 (0.2)a*	3.8 (0.19)	3.7 (0.22)
Total stress	67.7 (10.8)	18.6 (5.19)	19.2 (6.23)	88.9 (3.61)	31.1 (2.47)	28.2 (2.79)

(b)						
	PNS (*n* = 67)			NON-PNS (*n* = 673)		
Life events	Mean (SE)					
	Baseline	12 months	24 months	Baseline	12 months	24 months
Total dependent	12.3 (0.79)	5.1 (0.59)a***	4.6 (0.7)a***	15.4 (0.27)	7.8 (0.26)a***	7.6 (0.31)a***
Total independent	2.9 (0.22)	0.4 (0.15)a***	0.5 (0.18)a***	3.3 (0.07)	0.8 (0.07)a***	0.9 (0.08)a***
Total desirable	5.0 (0.28)	2.8 (0.25)a***	2.8 (0.28)a***	6.4 (0.09)	4.3 (0.10)a***	4.2 (0.1)a***
Total undesirable	8.1 (0.62)	2.6 (0.44)a***	2.2 (0.52)a***	9.9 (0.2)	3.8 (0.19)a***	3.7 (0.22)a***
Total stress	67.7 (10.8)	18.6 (5.19)a***	19.2 (6.23)a***	88.9 (3.61)	31.1 (2.47)a***	28.2 (2.79)a***

Mean represents the least squares means estimated by the generalized linear model, SE represents the standard error of the mean. a, significantly different from baseline; b, significantly different from 12 months; *p < 0.05, **p < 0.01, ***p < 0.001.

### 3.3. Differences in previous cannabis usage

Significantly more non-PNS group participants previously used cannabis (55.72%) compared to the PNS participants (35.82%, *x*^2^ = 11.10; *p* = 0.001). However, there were no significant differences between groups for the number of times cannabis was used in their lifetime, age of first usage, and current usage (Table 2).

**Table 2:** Rates and patterns of cannabis use over lifetime in PNS and non-PNS participants.

	non-PNS *n* = 673	PNS *n* = 67	Statistic	
	Mean (SD)	Mean (SD)	*t*	value of *p*
Number of times used in lifetime	128.7(125.95)	88.75 (114.19)	1.653	0.110
Age first tried	15.7 (2.80)	16.3 (3.2)	0.980	0.328
	*n* (%)	*n* (%)	*χ* ^2^	value of *p*
Current user: yes	154 (22.88)	9 (13.43)	0.034	0.855
Lifetime exposure: yes	375 (55.72)	24 (35.82)	11.10	0.001**

PNS, Persistent Negative Symptoms; SD, Standard Deviation.

### 3.4. Differences in resource utilization

There were no significant differences between the PNS and non-PNS groups for previous emergency visits due to physical problems, emergency visits for psychiatric problems, inpatient visits for physical problems, inpatient visits for psychiatric problems, nor day hospitalizations (*x*^2^ = 4.349; *p* = 0.500).

### 3.5. Differences in premorbid adjustment

**Figure 1:**
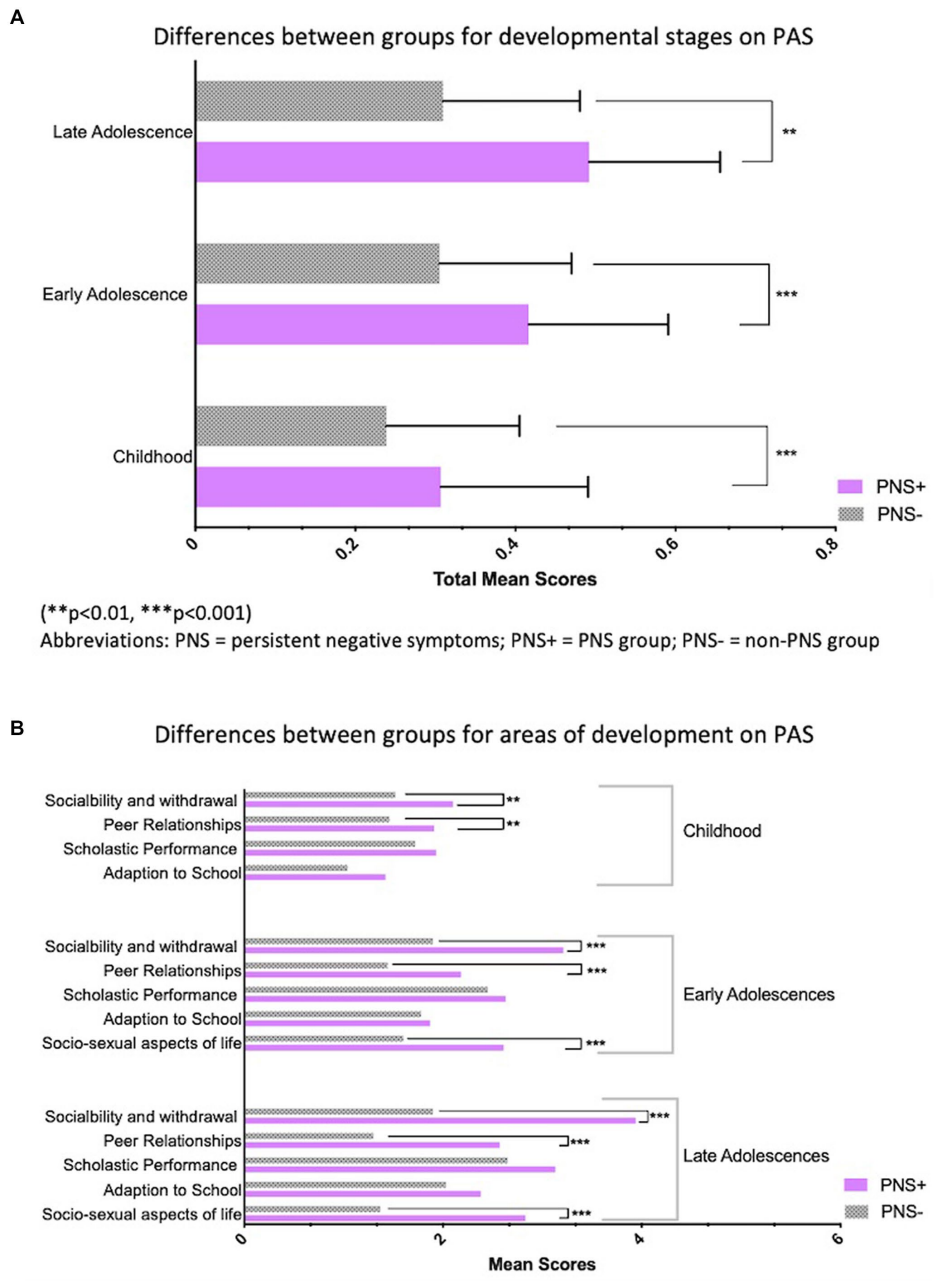
**(A)** Differences between group for developmental stages on PAS. (**p < 0.01, ***p < 0.001). PNS, persistent negative symptoms; PNS +, PNS group; PNS–, non-PNS group. **(B)** Differences between group for areas of developmental on PAS. (**p < 0.01, ***p < 0.001). PNS, persistent negative symptoms; PNS +, PNS group; PNS–, non-PNS group.

The PNS group had significantly poorer levels of premorbid adjustment in childhood (*M* = 0.31, SD = 0.02 vs. *M* = 0.24, SD = 0.007, *p* = 0.002), early adolescence (*M* = 0.42, SD = 0.02 vs. *M* = 0.31, SD = 0.007, *P* = <0.0001), and late adolescence (*M* = 0.49, SD = 0.02 vs. *M* = 0.31, SD = 0.008, *P* = <0.0001) compared to CHR participants without PNS, see Figures 1A,B.

### 3.6. Cluster analysis

**Figure 2:**
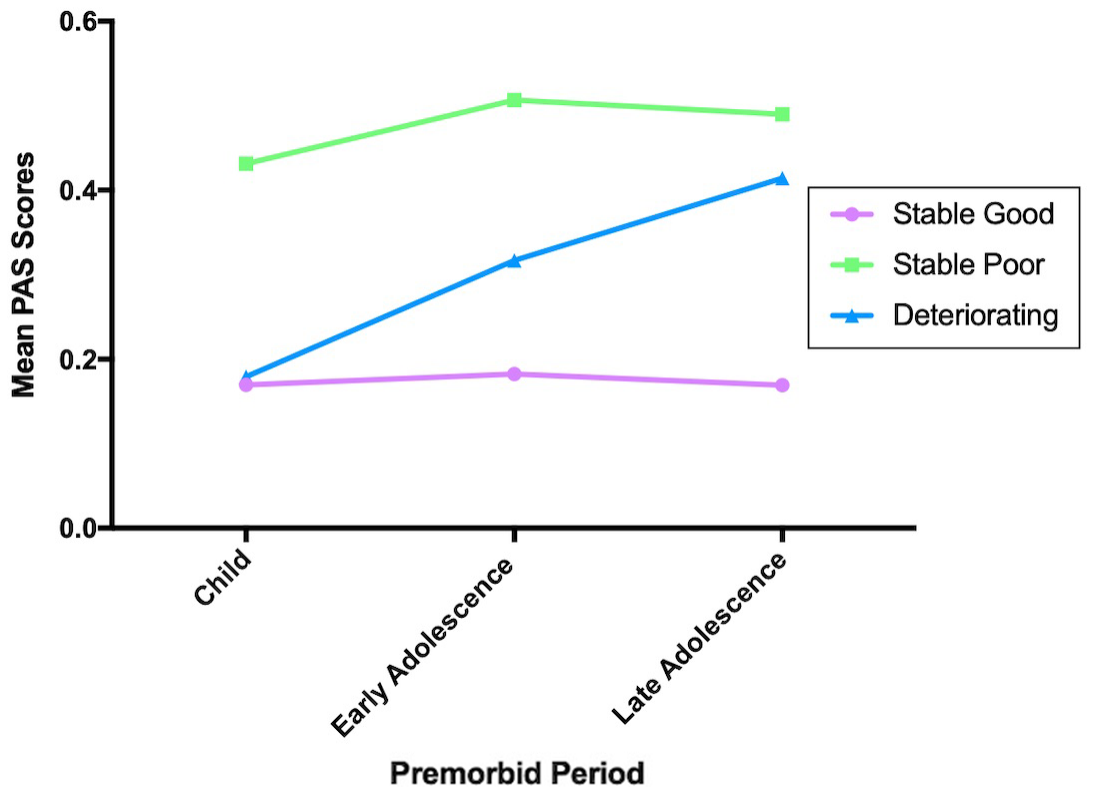
Premorbid clusters. For PAS scores higher score means poorer premorbid functioning.

Results of the cluster analyzes demonstrated that the best model was by pre-selecting three clusters versus selecting 2 or 4 clusters, which we labeled stable good (*n* = 285), stable poor (*n* = 196), and deteriorating (*n* = 228; see Figure 2). The greatest Euclidean distances were first between stable-good and stable-poor (0.53), followed by deteriorating and stable-poor (0.33), and deteriorating and stable-good (0.28). Chi-squared analysis demonstrated that there were significant differences among the clusters in terms of number of PNS participants (*x*^2^ = 33.68; *p* < 0.0001). There were more PNS individuals in the stable poor cluster and less PNS individuals in the stable good cluster than would have been expected by chance, with 56.7% of the PNS group in the stable poor cluster.

### 3.7. Prediction of PNS

For the prediction of PNS, a full logistic regression model was built with all explanatory variables including PAS total childhood, total early adolescence, total late adolescence, bullying and trauma. Variable inclusion and selection were made by dropping the variables with less significance one by one, creating a final model with one predictor (i.e., Total late adolescence). The significant logistic regression model (*χ*^2^ = 36.93, *p* < 0.0001) demonstrated that for a one-unit increase in total late adolescence the odds of being in PNS group are 405 times greater than the odds of being in non-PNS group.

## 4. Discussion

In summary, this paper examined PNS in a large CHR sample and their relationship with premorbid functioning, desirable and undesirable life events, trauma and bullying, previous cannabis use, and previous healthcare related resource utilization. There were no significant differences between the groups for trauma/bullying and healthcare related resource utilization. However, the non-PNS group reported significantly more cannabis usage and had significantly higher life events, both desirable and undesirable ones.

In terms of premorbid adjustment, the results demonstrated the PNS group had significantly poorer levels of premorbid adjustment across all three developmental periods, compared to the non-PNS group. Moreover, the cluster analyzes demonstrated distinct patterns of premorbid functioning. There were more PNS individuals in the stable poor cluster and less PNS individuals in the stable good cluster than would have been expected by chance, with 56.7% of the PNS group in the stable poor cluster. Finally, the logistic regression model indicated that poorer levels of premorbid adjustment in late adolescence significantly increased one’s odds of being in the PNS group compared to the non-PNS group.

The current results are consistent with previous studies which have shown that poorer premorbid functioning in late adolescence in CHR youth is significantly correlated with negative symptom severity (13, 14). One previous study that examined PNS in CHR youth found that the PNS group exhibited poorer premorbid functioning, overall and specifically that premorbid social adjustment was significantly poorer in the PNS group in both early and late adolescences, and academic adjustment in late adolescence (5). The results of the current study expand on this previous research, suggesting that not only does premorbid difficulties transverse the developmental periods for individuals with PNS, but that PNS is strongly predicted by poor premorbid adjustment in late adolescence. From the current cluster analysis, the observed trajectories of premorbid functioning in CHR youth are similar to previous results in CHR (34) and in patients with schizophrenia (35–37). Furthermore, our results are similar to Lyngberg et al. (13) and Horton et al. (38), in that the cluster analysis did not suggest a deteriorating group where CHR youth originate with poor premorbid functioning and continue to deteriorate.

Contrary to our hypothesis, we found no significant differences between the PNS and non-PNS groups for trauma and bullying. In patients with schizophrenia, several studies have documented associations between both trauma/bullying and negative symptoms (34, 39, 40). Specifically, early neglect has been associated with negative symptoms (39–41), and emotional neglect has been associated with greater negative symptoms severity (34). This is in contrast to the results of the current study and previous CHR studies, where no relationship between negative symptoms and trauma has been reported (16, 17). However, one CHR study did find an association between physical bullying and negative symptoms, albeit this was a very weak association (16).

Other notable points include that the non-PNS group reported significantly more cannabis usage, had significantly higher desirable and undesirable life events, and that there was no difference between the groups for healthcare resource utilization. In terms of life events, we predicted that undesirable life events would be greater in CHR youth with PNS, however it is possible that the non-PNS group have more motivation and have less anhedonia contributing to higher rates of life events regardless of type (i.e., desirable and undesirable life events). This confirms previous explanations that negative symptoms in CHR such as increased avolition and social withdrawal, may lead to fewer life events (18, 19). These results may also suggest that life events are not a contributing factor to later PNS in CHR youth. However, subjective recall differences between PNS and non-PNS group could explain life events differences particularly if PNS group had more expressive negative symptoms or fewer life events due to social withdrawal and amotivation.

In terms of cannabis usage, the current study found that more non-PNS group participants previously used cannabis compared to the PNS participants, with no significant differences between groups for the number of times cannabis was used in their lifetime, age of first usage, and current usage. These results are comparable to previous studies that have shown that total negative symptoms at baseline have not been linked with previous cannabis use (20–22). However, the results of the current study are contrary to another study that showed that current weekly cannabis users had higher total negative symptoms (23). Thus, the current results may suggest that cannabis use is not a contributing factor to later PNS in CHR youth.

Finally, the current study did not find any significant differences between the PNS and non-PNS groups for previous healthcare related utilization (i.e., emergency visits due to physical problems, emergency visits for psychiatric problems, inpatient visits for physical problems, inpatient visits for psychiatric problems, and day hospitalizations). In schizophrenia, negative symptoms have been showed to increased healthcare resource utilization, mainly in the primary care setting (3). Thus, the difference may be due to the current study not measuring other points of contact in primary care, such as visits to a family doctor, utilizing telehealth, or previous appointments with therapists.

### 4.1. Strengths and limitations

This study has several strengths, including a large longitudinal dataset to explore PNS and its association with early factors. However, a few limitations should be considered when interpreting the results of the current study. The first limitation is that we operationalized negative symptoms using the current domains of negative symptoms, which includes asociality, anhedonia, avolition, blunted affect, and alogia (2). Due to several limitations of measuring negative symptoms with the SOPS, we were only able to measure three areas of negative symptoms including social anhedonia, avolition, and expression of emotion, but no measure of asociality or alogia were examined. It is possible that if new CHR negative symptom scales are developed and validated to include the five negative symptom domains, future studies could measure more accurately the associations of PNS with early factors in CHR. One such scale that shows promise is the Negative Symptom Inventory-Psychosis Risk (NSI-PR), a scale developed specifically to measure the five domains of negative symptoms in CHR (42). Finally, other factors may have confounded this relationship such as depression and APS.

### 4.2. Directions for future research

The results of the current study may lead to some areas of future research. With the current focus of identification and treatment of CHR on attenuated positive symptoms, and a lack of efficacious treatments to help negative symptoms in CHR (43), an unfortunate course transpires for CHR youth with PNS who may not be identified as needing services in the first place and thus may not receive the assistance they need. To the best of our knowledge, no studies have examined the impact of interventions on PNS in a CHR sample. Thus, CHR researchers may wish to design future randomized control trials with a primary aim of impacting both negative symptoms and PNS. Secondly, it could be important to track other points of contact within the healthcare system in CHR youth, such as primary care visits, and determine if those with PNS use more primary care services which may be important to policy makers. Lastly, PNS research would benefit from scales that measure the five domains of negative symptoms and a consensus on how best to define PNS in CHR samples, this would help establish consistent PNS groups for targeted interventions and allow for consistent measuring when determining associations.

## 5. Conclusion

Results indicate that CHR youth with PNS have significantly lower levels of premorbid adjustment at all developmental periods compared to those without PNS. There were three patterns of premorbid functioning in our CHR sample, including stable good, stable poor and deteriorating. Those with PNS were overrepresented in the stable poor group. CHR youth with PNS may benefit from psychosocial treatments to address these deficits.

## Data availability statement

The raw data supporting the conclusions of this article will be made available by the authors, without undue reservation.

## Ethics statement

The studies involving human participants were reviewed and approved by both the University of Calgary and all other participating universities. All participants provided written informed consent, including parental consent.

## Author contributions

JA, TC, KC, BC, TM, DP, WS, MK, LS, MT, SW, EW, DM, and CB were responsible for the design of the study and for the supervisions of all aspects of data collection. DD and LL were responsible for the statistical analyzes. DD wrote the initial manuscript. All authors listed were involved in the study design and have contributed to and approved the final manuscript.

## Funding

This study was supported by the National Institute of Mental Health (grant U01 MH081984 to JA; grants U01 MH081928 and P50 MH080272; Commonwealth of Massachusetts SCDMH82101008006 to LS; grants R01 MH60720, U01 MH082022, and K24 MH76191 to KC; grant U01 MH081902 to TC; P50 MH066286 (Prodromal Core) to CB; grant U01 MH082004 to DP; grant U01 MH081988 to EW; grant U01 MH082022 to SW; and U01 MH081857-05 grant to BC). DD was funded by the Alberta Innovates Graduate Studentship.

## Conflict of interest

The authors declare that the research was conducted in the absence of any commercial or financial relationships that could be construed as a potential conflict of interest.

## Publisher’s note

All claims expressed in this article are solely those of the authors and do not necessarily represent those of their affiliated organizations, or those of the publisher, the editors and the reviewers. Any product that may be evaluated in this article, or claim that may be made by its manufacturer, is not guaranteed or endorsed by the publisher.
